# The Factors That Contribute to Dysfunctional Behavior in Active Military Personnel: An Umbrella Review

**DOI:** 10.1177/15248380251349776

**Published:** 2025-07-12

**Authors:** Jolene A. Cox, Scott McLean, Gemma J. M. Read, Paul M. Salmon

**Affiliations:** 1 Centre for Human Factors and Sociotechnical Systems, University of the Sunshine Coast, Sippy Downs, QLD, Australia; 2 School of Health, University of the Sunshine Coast, Sippy Downs, QLD, Australia

**Keywords:** contributory factors, dysfunctional behavior, military, systems thinking, unacceptable behavior

## Abstract

Dysfunctional behavior in active military personnel is a complex and challenging issue for military forces worldwide. Effective management of this issue requires a comprehensive understanding of the factors that contribute to dysfunctional behavior in military populations. The current study presents an umbrella review that synthesized and analyzed the existing literature on contributory factors to dysfunctional behavior in active military personnel using a systems thinking-based framework. Eleven systematic reviews were identified as eligible for inclusion in the umbrella review. The synthesis identified 14 contributory factors to the following types of dysfunctional behavior: suicidal behavior, substance misuse, domestic violence perpetration, and destructive leadership. The analysis indicated that existing literature focuses on contributory factors relating to the military personnel themselves and not influences in the broader military system or wider society. Additionally, few studies have sought to understand how factors interact to create dysfunctional behavior. Future research would benefit from the use of systems thinking-based frameworks and methods to investigate the factors, across the broader military system and society, that contribute to dysfunctional behavior in active military personnel.

Military forces worldwide exist for the main purpose of defending their country against domestic or foreign threats to the advancement of national security and prosperity (Australian Government Defence, n.d.a; U.S. Department of Defense, n.d.). The achievement of this national mission requires high-performing and cohesive teams of active military personnel. Thus, any personnel acting in a dysfunctional or unacceptable way can adversely affect not only military performance (e.g., reduce combat readiness) (Griffith, 2002) but also the health and well-being of those affected (Millegan et al., 2015; Shelef et al., 2024). In a military context, dysfunctional behavior can be defined as intentional acts that result in harm to the military personnel (themselves), other people (e.g., another military personnel, civilians, family members or partners), or the military service branch (Australian Government Defence, n.d.b). Examples of dysfunctional behavior include domestic or intimate partner violence, sexual assault and harassment, workplace bullying, suicide or self-harm, and substance misuse. The individual, organizational, economic, and societal costs of dysfunctional behavior in active military personnel have resulted in a growing interest in understanding and preventing this behavior.

Previous studies have estimated the prevalence of dysfunctional behavior in active military personnel, and these investigations have highlighted the extent of this issue. For example, Pruitt et al. (2019) reported that in 2015, the rate of suicide among U.S. active military personnel was 20.2 deaths per 100,000 personnel. Kelley et al. (2015) reported that approximately 25% of the U.S. Navy personnel who participated in their study (*N* = 108) indicated that they had perpetrated act(s) of violence toward their intimate partner in the previous 12 months. Moreau et al. (2021) found that in 2014 to 2015, nearly 40% of women and 20% of men among the French active military personnel surveyed (*N* = 1,500) reported experiencing military sexual trauma in the previous 12 months. More recently, the Commonwealth of Australia Royal Commission into Defence and Veteran Suicide (2024) indicated that in 2022, 38% of military personnel who responded to a Workplace Behaviour Survey (total *N* not reported) self-reported having experienced dysfunctional behavior in the previous 12 months. The reported prevalence across these studies suggests that dysfunctional behavior in active military personnel is a concern that needs addressing.

Effective management of dysfunctional behavior in active military personnel requires a comprehensive understanding of the factors that contribute to the various behaviors involved. Previous studies have found that experiences of suicidal ideation, thoughts, and behaviors before joining the military were associated with suicide attempts during military service (Bryan et al., 2014), poor mental health and experiences of substance problems were associated with suicide (LeardMann et al., 2013), a history of aggressive behavior before joining the military was associated with intimate partner violence perpetration (Stander et al., 2011), and hostility toward women and heavy drinking were associated with sexual harassment or assault perpetration (Stander et al., 2018). While this research has informed the knowledge base, a synthesis and analysis of the contributory factors across different types of dysfunctional behavior in active military personnel would be beneficial to further support the development of effective interventions. Taking a systems thinking-based approach can support such synthesis and analysis by informing what is known regarding the individual, organizational, and societal contributory factors to dysfunctional behavior in this military population. Of particular interest is whether different types of dysfunctional behavior have similar contributory factors that can be targeted when developing interventions (Salmon et al., 2020, 2022).

The discipline of systems thinking has been highly influential in studying complex problems (Leveson, 2004; Rasmussen, 1997). Advocates of systems thinking argue that when attempting to understand and address complex problems, a focus on individual components in isolation (e.g., individuals, tools, technologies, procedures) is limiting. Instead, they argue that overall complex systems should form the unit of analysis, enabling the study of how multiple components interact to influence behavior. This point is critical because, in complex systems, including those in the military context (Blouin, 2013; Surace, 2019), interactions among components are often nonlinear and difficult to predict, leading to the potential for unintended consequences (Cilliers, 1998). Another emphasis of systems thinking is that behaviors exhibited by frontline personnel are driven by broader system conditions, and hence, their behaviors should be treated as *consequences* rather than causes (Read, Shorrock, et al., 2021).

Rasmussen’s Risk Management Framework (RMF; Rasmussen, 1997) is one popular systems thinking-based framework that has been applied to examine complex public health issues. The RMF was developed initially to study the underlying causes of industrial safety accidents (Donovan et al., 2017; Goode et al., 2014; Read, Cox, et al., 2021; Salmon et al., 2010) but has been applied more broadly to study system behavior or performance across other domains, including in public health (Cox et al., 2024; Lane et al., 2020; Vicente & Christoffersen, 2006). According to the RMF, systems comprise several hierarchical levels (e.g., government, regulators, local government, service delivery, individuals, infrastructure) and stakeholders across these levels share the responsibility for the system’s behavior. The RMF is often used to understand, holistically, the network of factors that influence behavior by representing where factors reside in the system hierarchy (Svedung & Rasmussen, 2002). A recent example is the study by Cox et al. (2024), which aimed to understand the factors that influence help-seeking for self-harm in young people. Their analysis of the existing literature indicated that many of the known barriers to help-seeking for self-harm reside at lower levels of the help-seeking system hierarchy (i.e., service delivery and social environment, young people who self-harm). There was minimal knowledge of factors relating to the higher levels of the system (e.g., government, regulators) or the interactions among factors influencing help-seeking. The use of this systems thinking-based framework would also be highly beneficial in progressing our knowledge base on dysfunctional behavior in active military personnel.

The current study describes an umbrella review conducted to synthesize and analyze the existing literature on the factors that contribute to dysfunctional behavior in active military personnel. We chose to conduct an umbrella review over a systematic review as we were interested in the contributory factors across different types of dysfunctional behaviors rather than any specific type of dysfunctional behavior. The objectives of this umbrella review were to: (a) identify the known contributory factors to dysfunctional behavior in active military personnel and, using a systems thinking-based framework (RMF), (b) determine the extent to which previous research has studied these contributory factors from a systems thinking perspective.

## Method

### Protocol

This umbrella review was conducted according to the “Preferred Reporting Items for Overviews of Reviews (PRIOR) guidelines” (Gates et al., 2022). We developed a protocol describing the research aim and the planned methodology before conducting the review. We did not make any significant deviations from the protocol. Ethical approval was not required for the current study as our review did not involve recruiting or collecting data from human research participants.

### Electronic Search

We conducted the literature search in the period of May 14, 2024 to July 2, 2024 (inclusive), on three databases: Web of Science, Scopus, and PubMed. Based on our research aim and objectives, we developed Boolean search terms for “dysfunctional behavior” (broad and specific terms relating to different types of dysfunctional behavior) and “military personnel.” The terms were searched by title, abstract, and keywords in Web of Science and Scopus, and by title and abstract in PubMed. The search was limited to articles that were (a) published in the English language, (b) cataloged as reviews (Web of Science) or systematic reviews or meta-analyses (Scopus and PubMed), and (c) published in the last 10 years (i.e., 2014 up to the date of the final search). The final search string, the searched fields, and the search limits applied ensured that the initial search results contained only relevant articles and that the literature screening process would be manageable. The first author conducted the electronic search.

The final search string was as follows: ((((harm* OR unacceptable OR dysfunction* OR offen* OR destructive OR counterproductive OR devian*) AND (behavio* OR conduct OR act)) OR (sex* AND (assault OR harassment OR misconduct OR abuse)) OR (retaliation) OR ((domestic OR child) AND abuse) OR (bull* OR incivil*) OR ((workplace) AND (conflict)) OR (violen* OR aggress* OR battery OR assault) OR (suicid* OR “self harm” OR “self injur*” OR “self directed violence”) OR ((substance OR drug OR alcohol) AND (misuse OR abuse)) OR ((criminal OR illegal OR illicit) AND (behavio* OR conduct OR act)) OR (extremism) OR (violation)) AND (defen*e OR military OR “armed force*” OR servicem*n OR “serving personnel”)).

### Eligibility Criteria

We applied inclusion and exclusion criteria during the literature screening process to identify relevant articles.

#### Inclusion Criteria

We included articles in the review if they met the following criteria:

(a) The population focus included active military personnel (i.e., military personnel who were on active duty at the time of the study) from any country. There were no restrictions on the country of the population focus.
(b) The article focused on dysfunctional behavior in military personnel. For this review, we defined dysfunctional behavior as intentional acts that result in harm to the military personnel (themselves), other people (e.g., another military personnel, civilians, or the military personnel’s family members or partners), or the military service branch (Australian Government Defence, n.d.b). These behaviors may include, but not limited to, domestic or intimate partner violence, sexual assault and harassment, workplace bullying, suicide or self-harm, and substance misuse. A list of example behaviors considered, and their definitions can be found in Supplemental Material 1.
(c) The article must have identified, based on evidence from primary studies, contributory factors to dysfunctional behavior. For this review, we defined contributory factors as factors “that if it had not occurred or existed at the relevant time, then either the occurrence would probably not have occurred, adverse consequences associated with the occurrence would probably not have occurred or have been as serious, or another contributing safety factor would probably not have occurred or existed” (Australian Transport Safety Bureau, 2008).
(d) The article was a systematic review. We only considered the article to be a systematic review if the authors of the article specified conducting the review using recognized systematic review guidelines (e.g., Preferred Reporting of Items for Systematic Reviews and Meta-Analysis, Peer Review of Electronic Search Strategies).

#### Exclusion Criteria

We excluded articles from the review if they did not meet the inclusion criteria or any of the following criteria:

(a) The population focus did not include active military personnel or distinguish active military personnel from inactive military personnel (i.e., former-serving military personnel, veterans).
(b) The full-text version of the article was not available.
(c) The article was not a peer-reviewed journal article (e.g., preprints, conference proceedings, student theses, reports).
(d) The article was not published in the English language.
(e) The article was not published in the last 10 years (i.e., 2014 up to the date of the final search).

### Literature Screening Process

We used the web-based systematic review platform, Covidence, to support the literature screening process. After retrieving the initial search results, the first author removed article duplicates and screened the remaining articles against the eligibility criteria, first by title and abstract, and then by the full-text version. If there were insufficient details reported in an article to determine its relevance to the umbrella review, the research team discussed the article before making a consensus decision to include or exclude the article. The screening process resulted in the final set of articles (all systematic reviews) included in this umbrella review.

### Quality Assessment

We assessed the methodological quality of the included systematic reviews using the “A Measurement Tool to Assess Systematic Reviews 2” (AMSTAR-2) checklist (Shea et al., 2017). AMSTAR-2 is a validated instrument containing 16 items, covering domains such as literature searching and screening, assessing the risk of bias and the impact of bias on the interpretation and discussion of results, and reporting the conflicts of interest. Of the 16 items on the checklist, seven were critical items (items 2, 4, 7, 9, 11, 13, and 15). As per the checklist, systematic reviews with more than one critical flaw were rated as critically low quality, one critical flaw as low quality, more than one noncritical flaw as moderate quality, and no or one noncritical flaw as high quality. We did not consider the quality of systematic reviews in the eligibility criteria of our umbrella review, which was determined before our conduct of the review. As such, systematic reviews rated as low or critically low quality were not excluded from our review.

The first author and the third author conducted the quality assessment of the included systematic reviews independently. The level of agreement between the two raters was 92%. Disagreements were discussed and resolved via consensus.

### Corrected Covered Area Index

We assessed the degree of primary publication (i.e., a publication included in the systematic review) overlap in this umbrella review using the Corrected Covered Area (CCA) index (Hennessy & Johnson, 2020). In this context, overlap refers to the extent to which primary publications in the systematic reviews are the same or different. Determining the degree of overlap can support the analysis in the umbrella review and is recommended in umbrella review guidelines (Faulkner et al., 2022; Pollock et al., 2023). A high degree of overlap is considered problematic as it indicates that the same primary publications are included in multiple systematic reviews. However, a low degree of overlap may also be considered problematic, especially if it resulted from suboptimal search strategies. If deemed necessary, the issue of overlap can be addressed to minimize its influence on the analysis in the umbrella review (Lunny et al., 2021).

The CCA index was calculated using this mathematical formula: CCA = (*N* − *r*)/((*r* × *c*) − *r*), where *N* is the total number of times the primary publication was included in the systematic reviews (inclusive of double-counting), *r* is the number of unique primary publications, and *c* is the number of systematic reviews included in the umbrella review. The first author calculated the CCA index.

### Data Extraction and Presentation

We extracted the following information from each included systematic review: lead author name and year of publication, the dysfunctional behavior studied, military populations included, military service branch included, the number of primary publications included in the systematic review, the number of primary publications relevant to this umbrella review, contributory factor(s) to dysfunctional behavior in active military personnel, sociodemographic factor(s), and any specified relationships between contributory factors.

The extraction of contributory factors, sociodemographic factors, and relationships between contributory factors was based on our review and interpretation of the results and discussion sections of the included systematic reviews. After data extraction, the contributory factors were mapped onto a generic military system hierarchy, adapted from Rasmussen’s RMF (Rasmussen, 1997). The generic military system hierarchy has the following six levels:

(a) *Government*: The government and government agencies that play a role in developing and maintaining military forces and conducting military operations.
(b) *Regulatory bodies and associations*: Regulatory bodies and associations that play a role in ensuring the safety, health and well-being, or performance of military forces (e.g., insurers, safety regulators, advocacy bodies, universities).
(c) *Organization management, planning, and budgeting*: Organizations and individuals who play a role in managing, planning, and budgeting of military forces (e.g., director general, senior executive, Chief of Army).
(d) *Supervision, management, and social environment of military personnel*: Individuals who play a role in supervising and managing military personnel (e.g., commanders, work supervisors, colleagues) or individuals who make up the social environment of military personnel (e.g., family, partner, friends).
(e) *Military personnel and processes*: Military personnel and the personnel’s knowledge, skills, decisions, actions, backgrounds, and experiences.
(f) *Physical equipment, infrastructure, and surroundings*: The physical equipment, infrastructure, and surroundings of the military environment with which military personnel interact.

To illustrate how we extracted contributory factors: If a systematic review identified “moral injury experiences” as a contributory factor to a dysfunctional behavior, the factor was placed at the *Military personnel and processes* level of the system, as this factor relates to the military personnel’s experiences of an injury to their moral conscience and values. If a systematic review identified “deployment” as a contributory factor, the factor was placed at the *Government* level, as this factor relates to the movement of military forces to pursue specific military objectives. Additionally, a contributory factor was placed across two or more levels if the factor related to the actions and decisions of stakeholders across these multiple levels. For example, the factor “family relationship problems” was placed across the *Military personnel and processes* level and *Supervision, management, and social environment of military personnel* level, as this factor relates to the conflicts and disagreements between both the military personnel (who reside at the *Military personnel and processes* level) and their family (who reside at the *Supervision, management, and social environment of military personnel* level).

The first author and the second author extracted data across the included systematic reviews independently. Disagreements were discussed and resolved via consensus.

## Results

### Full-text Selection

**Figure 1.**
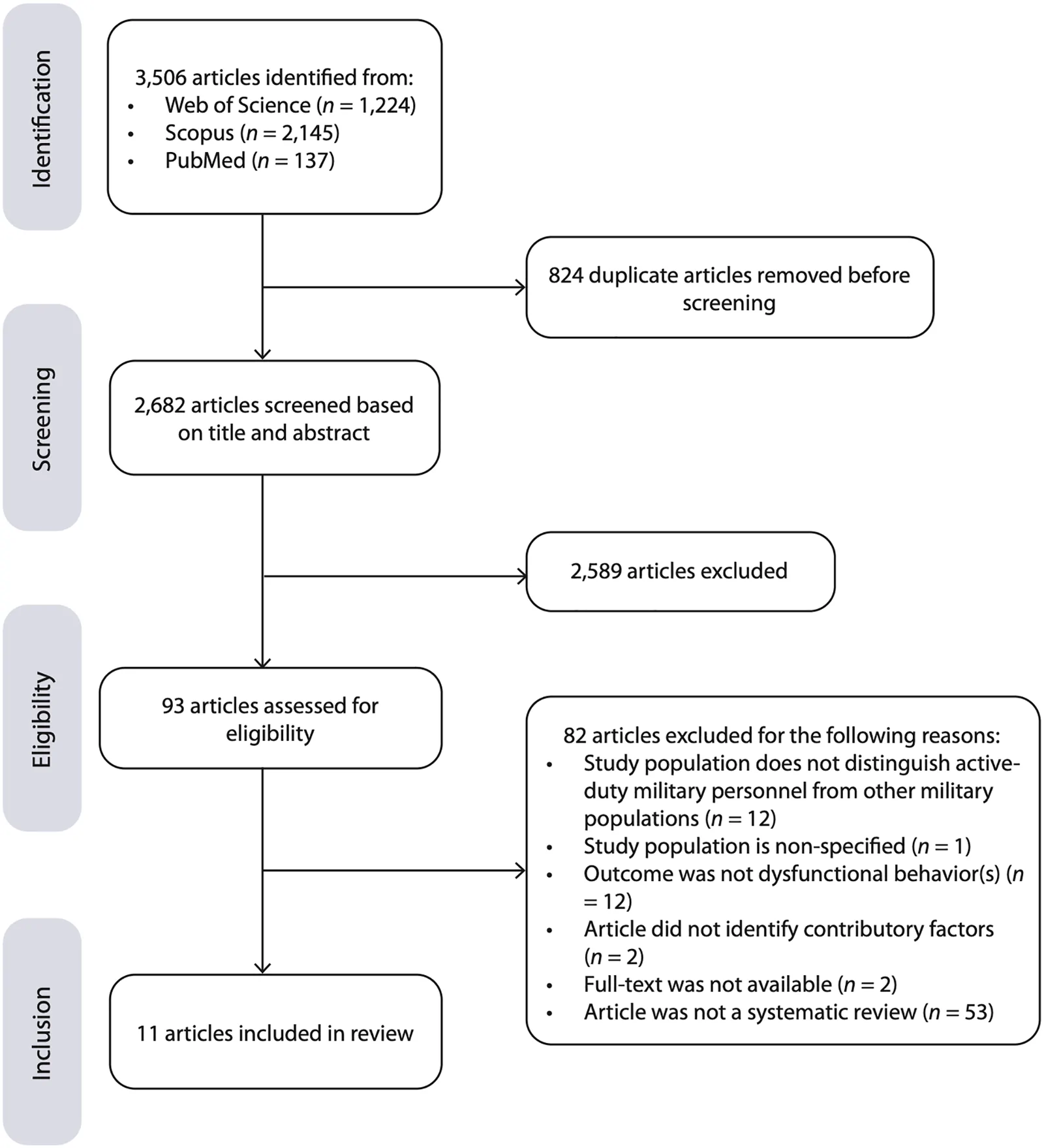
Flowchart of the umbrella review screening process.

The process of our full-text selection can be found in Figure 1. The literature search retrieved a total of 3,506 articles. Of these articles, 824 were identified as duplicates and excluded from the umbrella review. The title and abstract of the remaining 2,682 articles were screened against the eligibility criteria. This resulted in the exclusion of 2,589 articles. The full-text version of the remaining 93 articles was screened, resulting in a further 82 articles being excluded (see Supplemental Material 2 for reasons behind the exclusions). The entire screening process resulted in the inclusion of 11 systematic review articles in the umbrella review (Forkus et al., 2021; Fosse et al., 2019; Hall et al., 2022; Holliday et al., 2020; Jamieson et al., 2023; Kelsall et al., 2015; Kwan et al., 2020; McKenzie et al., 2023; Trevillion et al., 2015; Williamson et al., 2024; Yancey et al., 2024).

### Quality Assessment

The majority of the included systematic reviews (82%) were rated as critically low quality, with the remaining (18%) rated as low quality (see Supplemental Material 3 for ratings of individual items in AMSTAR-2). The main methodological limitations included the absence of an explicit statement that the review methods were established before conducting the systematic review (Item 2) and information on why studies were excluded from the systematic review (Item 7).

### CCA Index

There was minimal overlap of primary publications across the included systematic reviews (0.89%). The calculated CCA index was: (*N* − *r*)/((*r* × *c*) − *r*) = (49 − 45)/((45 × 11) − 45) = 0.0089. We did not consider this minimal overlap as an issue requiring addressing, as it is likely a reflection of the search limits (e.g., systematic reviews published in the last 10 years), eligibility criteria of the umbrella review, and the breadth of dysfunctional behavior examined in this review.

### Publication Information

The publication information extracted from the included systematic reviews can be found in Table 1. The systematic reviews were published between 2015 and 2024. The most frequently studied behavior was suicidal behavior (suicide or suicide attempts; *n* = 6), which is defined as any fatal or nonfatal acts of intentionally ending one’s own life (Nock et al., 2008). Other behaviors include substance misuse (*n* = 4), defined as an excessive or inappropriate use of drugs or alcohol (McLellan, 2017), domestic violence perpetration (*n* = 1), defined as any form of violence toward others within a family or domestic context (Australian Institute of Health and Welfare, 2025), and destructive leadership (*n* = 1). Destructive leadership is broadly defined as systemic and repeated behavior by a leader, supervisor, or manager that violates organizational interests by undermining and/or sabotaging the organization’s goals, tasks, and resources, and staff’s job satisfaction and their well-being (Einarsen et al., 2007).

**Table 1.** Publication Information of the Included Systematic Reviews.

Citation	Dysfunctional Behavior Type Studied	Military Populations Included	Military Service Branch	Number of Primary Publications Included	Number of Primary Publications Relevant to Umbrella Review	AMSTAR-2 Rating
Forkus et al. (2021)	Substance misuse	Active duty Inactive	Nonspecific National Guard Reserves	47	5	Critically low
Fosse et al. (2019)	Suicidal behavior	Active duty	Nonspecific National Guard Reserves	Qualitative synthesis: 27 Quantitative synthesis: 22	5	Critically low
Hall et al. (2022)	Destructive leadership	Active duty Inactive Nonmilitary	Nonspecific National Guard	Qualitative synthesis: 57 Quantitative synthesis: 49	4	Critically low
Holliday et al. (2020)	Substance misuse	Active duty Inactive	Nonspecific National Guard Reserves	48	6	Low
Jamieson et al. (2023)	Suicidal behavior	Active duty Inactive	Nonspecific	12	1	Critically low
Kelsall et al. (2015)	Suicidal behavior	Active duty Inactive	Nonspecific National Guard Reserves	Qualitative synthesis: 18 Quantitative synthesis: 16	8	Critically low
Kwan et al. (2020)	Suicidal behavior	Active duty Inactive	Nonspecific National Guard Reserves	Qualitative synthesis: 42 Quantitative synthesis: 28	7	Critically low
McKenzie et al. (2023)	Substance misuse	Active duty Inactive	Nonspecific National Service	83	7	Critically low
Trevillion et al. (2015)	Intimate partner violence perpetration	Active duty Inactive	Nonspecific National Guard	10	2	Low
Williamson et al. (2024)	Suicidal behavior	Active duty Inactive	Service branch not reported Reserves	28	1	Critically low
Yancey et al. (2024)	Domestic violence perpetration	Active duty Inactive	Not reported	43	1	Critically low

*Note.* AMSTAR-2 = A Measurement Tool to Assess Systematic Reviews 2.

### Contributory Factors

**Figure 2.**
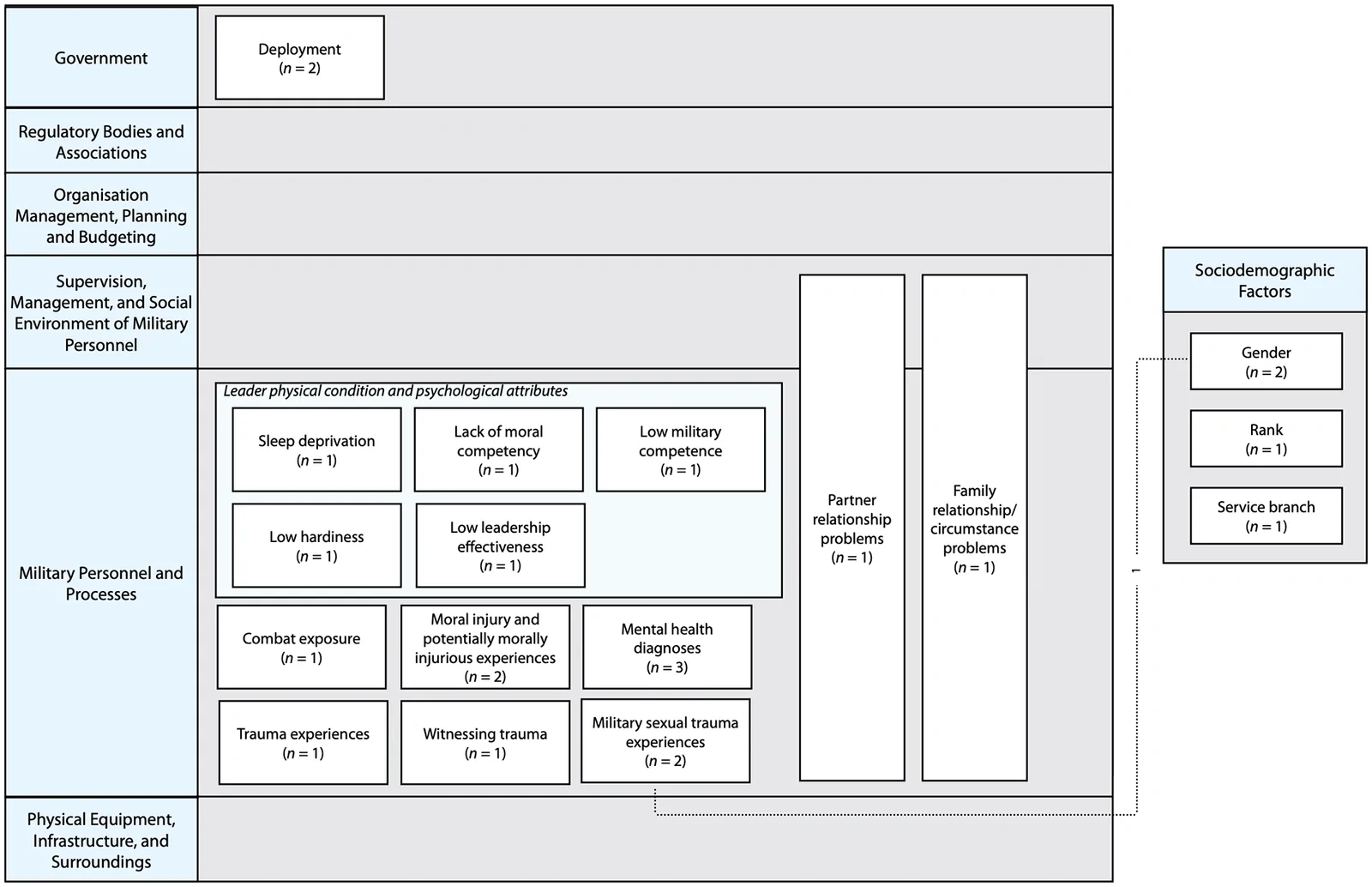
Dysfunctional behavior contributory factors across the military system. *Note.* The number of studies from which the contributory factor or sociodemographic factor was extracted is indicated by the number in parentheses. Relationships among factors are indicated by the lines linking the factors, and the number of studies from which the relationships were extracted is indicated on the lines (note that only one relationship was identified in this umbrella review).

The contributory factors, descriptions of these factors, the level where they reside in the military system, and the corresponding dysfunctional behavior can be found in Table 2. The mapping of the contributory factors across the military system is presented in Figure 2.

**Table 2.** Dysfunctional Behavior Contributory Factors Extracted From the Included Systematic Reviews.

Factor	Definition	System Level	Dysfunctional Behavior
Deployment	Movement of military personnel to operations within the military personnel’s home country or overseas.	Government	Substance misuse (Kelsall et al., 2015) Suicidal behavior (Williamson et al., 2024)
Partner relationship problems	Conflicts or disagreements between the military personnel and their partner.	Supervision, management, and social environment of military personnel	Suicidal behavior (Holliday et al., 2020)
Family relationship/circumstance problems	Conflicts or disagreements between the military personnel and their family, or adverse family circumstances.	Military personnel and processes	Suicidal behavior (Holliday et al., 2020)
Leader attributes			
Sleep deprivation	A physical and mental state resulting from not having adequate duration and/or quality of sleep.	Supervision, management, and social environment of military personnel	Destructive leadership (Fosse et al., 2019)
Lack of moral competency	The inability to problem-solve and judge moral issues in an unbiased way.	Supervision, management, and social environment of military personnel	Destructive leadership (Fosse et al., 2019)
Low military competence	A poor ability to develop and demonstrate the skills of military leaders to lead their subordinates.	Supervision, management, and social environment of military personnel	Destructive leadership (Fosse et al., 2019)
Low hardiness	A poor ability to manage and respond to challenging or stressful events.	Supervision, management, and social environment of military personnel	Destructive leadership (Fosse et al., 2019)
Low leadership effectiveness	A poor ability to influence a team or a group of people.	Supervision, management, and social environment of military personnel	Destructive leadership (Fosse et al., 2019)
Military sexual trauma experiences	The experience of a sexual assault during military service.	Military personnel and processes	Substance misuse (Forkus et al., 2021; Yancey et al., 2024) Suicidal behavior (Forkus et al., 2021)
Moral injury and potentially morally injurious experiences	A cognitive and emotional response that results from events that violate the military personnel’s moral conscious and values.	Military personnel and processes	Substance misuse (Hall et al., 2022) Suicidal behavior (Hall et al., 2022; Jamieson et al., 2023)
Mental health diagnoses	A diagnosis of a mental health condition that affects the military personnel’s mood, thinking, and behavior.	Military personnel and processes	Domestic violence perpetration (Trevillion et al., 2015) Suicidal behavior (Holliday et al., 2020; McKenzie et al., 2023)
Trauma experiences	The experience of trauma (toward oneself) during military service.	Military personnel and processes	Suicidal behavior (Williamson et al., 2024)
Witnessing trauma	The witnessing of trauma during military service.	Military personnel and processes	Suicidal behavior (Williamson et al., 2024)
Combat exposure	The exposure to traumatic events during military service.	Military personnel and processes	Substance misuse (Yancey et al., 2024)

A total of 14 contributory factors were extracted from the included systematic reviews. Across the military system, one contributory factor was mapped to the *Government* level, two at the *Supervision, management, and social environment of the military personnel* level, and 13 at the *Military personnel and processes* level (note that 2 contributory factors were mapped across both the *Supervision, management, and social environment of the military personnel* level and the *Military personnel and processes* level due to shared decisions and actions). None of the identified contributory factors was mapped to the *Regulatory bodies and associations, Organization management, planning, and budgeting* or *Physical equipment, infrastructure, and surroundings* levels. The most frequently reported contributory factor across the included systematic reviews was mental health diagnoses (*n* = 3), followed by deployment (*n* = 2), moral injury and potentially morally injurious experiences (*n* = 2), and military sexual trauma experiences (*n* = 2).

### Sociodemographic Factors

The sociodemographic factors extracted from the included systematic reviews are presented in Figure 2. Only three sociodemographic factors were extracted: Gender (*n* = 2), military rank (*n* = 1), and service branch (*n* = 1).

### Relationships Between Factors

Only one relationship between factors was reported in the included articles. This was a relationship between military sexual trauma experiences and a sociodemographic factor, gender (*n* = 1). Females were more likely than males to report experiencing military sexual trauma (Maguen et al., 2012, as cited in Forkus et al., 2021).

## Discussion

Dysfunctional behavior in active military personnel is a complex and challenging issue faced by military forces worldwide. Such behavior can significantly impact the health and well-being of the people involved (including the military personnel themselves) and the performance of military forces. As there are various types of dysfunctional behavior, a comprehensive understanding of contributory factors is needed to support the development of effective prevention strategies. In particular, understanding systemic and societal contributory factors may support the development of strategies that can address multiple types of dysfunctional behavior rather than one in isolation. The aim of our umbrella review was to (a) identify the contributory factors to dysfunctional behavior in active military personnel and (b) determine the extent to which previous research has studied these contributory factors from a systems thinking perspective. Our study represents the first in the field to take a systems thinking-based approach to this complex issue. A summary of the critical findings can be found in Table 3.

**Table 3.** Critical Findings From the Umbrella Review.

• There is a need to conduct systematic reviews on contributory factors to a wider range of dysfunctional behavior, including sexual assault and harassment, bullying and incivility, and extremism, in active military personnel. • Many of the known contributory factors extracted in this umbrella review were related to the military personnel involved and their knowledge, skills, decisions, actions, and experiences. More research is needed to understand how decisions and actions from the broader military system and wider society contribute to dysfunctional behavior in active military personnel. This research field will benefit from the use of systems thinking-based frameworks and methods to investigate the societal and military system factors that contribute to dysfunctional behavior in active military personnel.

Across the 11 included systematic reviews, only four types of dysfunctional behaviors were studied (in order of frequency): suicidal behavior (suicide or suicide attempts), substance misuse, domestic violence perpetration, and destructive leadership. This highlights the critical need for systematic reviews of other types of dysfunctional behavior to be conducted, including sexual assault and harassment, bullying and incivility, and extremism. The most frequently reported contributory factors to dysfunctional behavior included mental health diagnoses, deployment, moral injury and potentially morally injurious experiences, and military sexual trauma experiences. Our analysis indicated that many of the known contributory factors were related to the military personnel involved and their knowledge, skills, decisions, actions, and experiences (*Military personnel and processes* level). For example, having mental health difficulties that led to a diagnosis contributed to domestic violence perpetration (Trevillion et al., 2015) and suicidal behavior (Holliday et al., 2020; McKenzie et al., 2023), the experience of trauma (toward oneself) during military service contributed to suicidal behavior (Williamson et al., 2024), and the witnessing of trauma toward others during service also contributed to suicidal behavior (Williamson et al., 2024). Certain leadership physical conditions and psychological attributes of military personnel, such as sleep deprivation, lack of moral competence, and low hardiness, were also among the known contributory factors to their dysfunctional behavior (destructive leadership, specifically) (Fosse et al., 2019). Our analysis only found one contributory factor related to the decisions and actions of government and government bodies (*Government* level)—military deployment. Only one relationship between factors was identified: between military sexual trauma experiences and the sociodemographic factor, gender. Overall, our findings suggest that the existing research on dysfunctional behavior in active military personnel has *disproportionately* focused on the attributes or characteristics of the military personnel themselves (individual-level factors) and not on other systemic factors that may influence the engagement in dysfunctional behavior. Additionally, existing research has not investigated the relationships or interactions between contributory factors that influence dysfunctional behavior. Both gaps in the knowledge base are critical, suggesting that a systems thinking perspective has not yet been adopted, and that there is a limited understanding of the complex set of factors that interact to influence active military personnel’s engagement in dysfunctional behavior.

The disproportionate focus on individual-level factors contributing to behavior is a common finding in reviews that have used a systems thinking-based approach (Cox et al., 2024; Lane et al., 2020; Read, Cox, et al., 2021). Importantly, this focus frames individuals as solely responsible for their behavior. The tendency to overattribute responsibility to individuals has been especially emphasized in health behaviors, such as obesity (Rodhain & Gourmelen, 2018) and mental health (Foster & O’Mealey, 2022), and has implicated the extent of policy support to address these behaviors (Temmann et al., 2021). This tendency is also observed in dysfunctional behavior. For example, the Australian Government Defence policy on behavioral standards required of all Defence Force personnel (Instruction PPL7—Required Behaviours in Defence) states that personnel “are personally responsible for their behaviour . . .” and “are responsible for questioning behaviour that could reasonably be interpreted as unaccepted.” Consequently, the Australian Government Defence responds to dysfunctional behavior with behavior training for their recruits (Commonwealth Ombudsman, 2020; Cronshaw, 2023). Their current policy and response ignore the role of systemic military and societal factors in contributing to dysfunctional behavior. From a theoretical point of view, the absence of evidence that systemic factors beyond individual-level factors contribute to dysfunctional behavior should not be taken as evidence of absence. Instead, the absence of evidence highlights a critical knowledge gap, describing the lack of research on how systemic factors (e.g., decisions, actions, and conditions in the broader military or society) contribute to dysfunctional behavior. This knowledge gap also indicates that existing research has overlooked key systems thinking tenets; specifically, that behavior emerges from the interactions among stakeholders at all levels of the system and that stakeholders across the system share responsibility for the emergent behavior. As such, we strongly recommend that the current focus on individual military personnel characteristics and attributes as *causes* of dysfunctional behavior be shifted to instead view these characteristics and attributes as *consequences* of other factors residing in military systems and society. Future research should strive to identify these factors and how they interact with each other to influence the behavior of active military personnel. This can be achieved with a comprehensive systems thinking-based study of the individual-level and systemic factors across the military system that contribute to dysfunctional behavior in military personnel.

The widespread applications of the RMF to study the causes of safety accidents have allowed Salmon et al. (2020) to investigate whether there is a set of common, recurring contributory factors and interrelations that can be applied across different disciplines. Their review of 23 analyses using the RMF reduced 5,587 distinct contributory factors into a “common causal network” that may contribute to adverse events across domains. This included various systemic factors such as personnel management and recruitment processes, the judgment and decision-making of regulatory bodies and associations and the government, the standards, policies, and regulations developed and implemented by regulatory bodies and associations, and the political structures and services of the government. Notably, similar contributory factors were not identified from existing research in our review, but they are likely to apply to dysfunctional behavior in military personnel also. It is worth noting that the review by Salmon et al. (2020) was focused on common, recurring contributory factors to safety accidents, reflecting the historical use of RMF. As the framework is increasingly being used to study the causes or drivers of public health issues, it would be useful for the field to conduct a similar review in the near future, investigating whether there is a set of contributory factors that could apply to the study of any public health issue. Identifying a set of common contributory factors to behavior across a wide range of complex systems and system outcomes can support future analyses and prevention or intervention activities.

Our umbrella review demonstrates the value of using the RMF to enhance our knowledge base on dysfunctional behavior in active military personnel. While we encourage researchers to adopt this systems thinking-based framework to study other complex systems, researchers should also be aware of other similar frameworks before deciding on the framework that is most appropriate for their study. An example of another framework is the Applied Pragmatic Functional Contextualism (APFC; Cwinn & Hamel, 2024), which was recently proposed as a theoretical framework designed specifically for applied mental health research. This new theoretical framework serves as an alternative approach to conventional mental health research, where content-based theoretical approaches are often relied upon. Like the RMF, the APFC emphasizes the importance of the contextual and systemic factors that impact behavior. We believe that researchers should consider adopting systems thinking-based approaches in their future research to support a more holistic understanding of dysfunctional behavior in active military personnel and other mental health topics more broadly.

Findings from our umbrella review should be considered within the constraints and limitations of the review. A constraint of note is the population focus of the review. We chose to include systematic reviews on dysfunctional behavior in *active* military personnel and exclude those that did not include active military personnel or distinguish findings related to active military personnel from inactive military personnel (former-serving military personnel or veterans). This decision was based on the observation that the prevalence of dysfunctional behavior in active military personnel is increasing worldwide and that the contributory factors to dysfunctional behavior in active military personnel are likely to differ from those in inactive military personnel (e.g., reasons for discharge from military service). This eligibility criterion resulted in fewer systematic reviews being included in our umbrella review for synthesis and analysis. In our screening process, it was evident that previous military-related research has often studied inactive military personnel and not active military personnel. While it is important to understand the impacts of military service on inactive military personnel, it is equally important to understand the impacts on active military personnel, especially since this research will inform current military operations and policy. A consideration of the umbrella review is the limited analysis on gender or cultural diversity within active military populations. The included systematic reviews were consistent in their reporting of gender and ethnic backgrounds of the military populations in the primary studies; however, many of these systematic reviews were not focused on gender or ethnic differences in their analyses. As a result, our analysis identified only two systematic reviews that reported gender differences in contributory factors to dysfunctional behavior (Forkus et al., 2021; Kwan et al., 2020). We encourage researchers to consider gender and ethnic diversity in their study of military populations to improve inclusivity and generalizability of military-related research. Another consideration of the umbrella review is the methodological quality of the included systematic reviews. Our quality assessment (using AMSTAR-2) indicated that all 11 included systematic reviews were considered to have critically low or low methodological quality, potentially affecting the findings from our umbrella review. We chose not to exclude systematic reviews based on their methodological quality. Had we done so, none of the systematic reviews retrieved from our literature search and screening process would be eligible for synthesis and analysis in our umbrella review. The problem of poor methodological quality of systematic reviews appears to be pervasive across different disciplines. For example, Li et al. (2022) reported that in their assessment of 81 eligible systematic reviews in heart failure-related research (using AMSTAR-2), 79 studies were of critically low quality and 2 were of low quality. Bojcic et al. (2024) reported similar observations in their recent cross-sectional meta-research study on the methodological quality of systematic reviews. As systematic reviews are often relied upon to provide an accurate summary of available evidence, we encourage researchers conducting systematic reviews to self-assess their protocols using AMSTAR-2 to ensure that their planned reviews will meet the requirements of a high or moderate methodological quality.

## Conclusion

This umbrella review synthesized the existing literature on dysfunctional behavior in active military personnel, and using a systems thinking-based framework, analyzed the known contributory factors to this behavior. Our synthesis and analysis indicate that previous studies have focused on contributory factors related to the military personnel themselves, with minimal consideration of the factors relating to the broader military system and society. It is our view that more comprehensive systems thinking-based investigations of contributory factors to dysfunctional behavior in active military personnel should be conducted. The proposed research will enhance our knowledge base on the causes of dysfunctional behavior in active military personnel and, as a result, support the development of more effective prevention strategies to address this complex issue. Implications for research, practice, and policy can be found in Table 4.

**Table 4.** Implications for Research, Practice, and Policy.

Implications	
Research	• Future research should consider the influences of contributory factors beyond the military personnel involved that is, factors in the military system and society. • Future research should investigate the relationships or interactions between contributory factors that influence dysfunctional behavior in this population.
Practice	• In order to develop effective preventative strategies, military forces worldwide should invest in further research to develop a comprehensive understanding of contributory factors to dysfunctional behavior in active military personnel.
Policy	• Policy and preventative activities need to consider the role of systemic military and societal factors in contributing to dysfunctional behavior in active military personnel.

## Supplemental Material

sj-docx-1-tva-10.1177_15248380251349776 – Supplemental material for The Factors That Contribute to Dysfunctional Behavior in Active Military Personnel: An Umbrella Review Supplemental material, sj-docx-1-tva-10.1177_15248380251349776 for The Factors That Contribute to Dysfunctional Behavior in Active Military Personnel: An Umbrella Review by Jolene A. Cox, Scott McLean, Gemma J. M. Read and Paul M. Salmon in Trauma, Violence, & Abuse

sj-docx-2-tva-10.1177_15248380251349776 – Supplemental material for The Factors That Contribute to Dysfunctional Behavior in Active Military Personnel: An Umbrella Review Supplemental material, sj-docx-2-tva-10.1177_15248380251349776 for The Factors That Contribute to Dysfunctional Behavior in Active Military Personnel: An Umbrella Review by Jolene A. Cox, Scott McLean, Gemma J. M. Read and Paul M. Salmon in Trauma, Violence, & Abuse

sj-docx-3-tva-10.1177_15248380251349776 – Supplemental material for The Factors That Contribute to Dysfunctional Behavior in Active Military Personnel: An Umbrella Review Supplemental material, sj-docx-3-tva-10.1177_15248380251349776 for The Factors That Contribute to Dysfunctional Behavior in Active Military Personnel: An Umbrella Review by Jolene A. Cox, Scott McLean, Gemma J. M. Read and Paul M. Salmon in Trauma, Violence, & Abuse
