# Supplementary material for: The Factors That Contribute to Dysfunctional Behavior in Active Military Personnel: An Umbrella Review
Source: Trauma Violence Abuse. 2025 Jul 12;27(4):1448–61. doi: 10.1177/15248380251349776 (PMC13578646; doi:10.1177/15248380251349776)
Supplement: Supplementary file 1 [file sj-docx-1-tva-10.1177_15248380251349776.docx]

# Supplemental Material 1

**Table S1**

Examples of Dysfunctional Behaviors Considered in the Umbrella Review

| Behavior | Definition |
| --- | --- |
| Destructive leadership | The systemic and repeated behavior by a leader, supervisor, or manager that violates organizational interests by undermining and/or sabotaging the organization’s goals, tasks, and resources, and staff’s job satisfaction and their well-being (Einarsen et al., 2007). |
| Domestic violence perpetration | Any form of violence towards others within a family or domestic context (Australian Institute of Health and Welfare, 2025). This includes intimate partner violence perpetration. |
| Extremism | Any unlawful acts to support the use of violence by others to promote a political, ideological, or religious goal (Council of Australian Governments, 2015). |
| Bullying | A persistent and unreasonable behavior directed at a person that creates a risk to their health and safety (Smith, 2016). |
| Incivility | A low-intensity behavior that is rude, disruptive, and disrespectful, intended to cause discord and disharmony (Jackson et al., 2024; Wilson et al., 2023). |
| Retaliation | A harmful act committed in response to an actual or perceived harm (Legal Information Institute, 2020). |
| Sexual misconduct | Any inappropriate or unwanted sexual act or behavior that a person does not consent to. This may include sexual assault, sexual harassment, or sexual abuse (National Sexual Violence Resource Center, n.d.). |
| Substance misuse | An excessive or inappropriate use of drugs or alcohol (McLellan, 2017). |
| Suicidal behavior | A fatal or non-fatal act of intentionally ending one’s own life (Nock et al., 2008). |

*Note.* In the umbrella review, dysfunctional behavior was defined as intentional acts that result in harm to the military personnel (themselves), other people (e.g., another military personnel, civilians, family members or partners), or the military service branch (Australian Government Defence, n.d.-b).

**References**

Australian Government Defence. (n.d.-b). *Unacceptable behaviour*. <https://www.defence.gov.au/about/complaints-incident-reporting/unacceptable-behaviour>

Australian Institute of Health and Welfare (2025). Understanding family, domestic and sexual violence. <https://www.aihw.gov.au/family-domestic-and-sexual-violence>

Council of Australian Governments (2015). Australia’s Counter-Terrorism Strategy. <https://www.nationalsecurity.gov.au/what-australia-is-doing-subsite/Files/australias-counter-terrorism-strategy-2015.pdf>

Einarsen, S., Aasland, M. S., & Skogstad, A. (2007). Destructive leadership behaviour: A definition and conceptual model. *The Leadership Quarterly, 18*(3), 207–216. <https://doi.org/10.1016/j.leaqua.2007.03.002>

Jackson, D., Usher, K., & Cleary, M. (2024). Workplace incivility: Insidious, pervasive and harmful. *International Journal of Mental Health Nursing, 33*(3), 483–486. <https://doi.org/10.1111/inm.13315>

Legal Information Institute (2020). Retaliation. <https://www.law.cornell.edu/wex/retaliation>

McLellan, A. T. (2017). Substance misuse and substance use disorders: Why do they matter in healthcare? *Transactions of the American Clinical and Climatological Association, 128,* 112–130.

National Sexual Violence Resource Center (n.d.). Let’s Talk: Definitions of terms. <https://www.nsvrc.org/lets-talk-campus/definitions-of-terms>

Nock, M. K., Borges, G., Bromet, E. J., Cha, C. B., Kessler, R. C., & Lee, S. (2008). Suicide and suicidal behavior. *Epidemiologic Reviews, 30*(1), 133–154. <https://doi.org/10.1093/epirev/mxn002>

Smith, P. K. (2016). Bullying: Definition, types, causes, consequences and intervention. *Social and Personality Psychology Compass, 10*(9), 519–532. <https://doi.org/10.1111/spc3.12266>

Wilson, S.T., Urban, R.W. & Smith, J.G. (2023) Online prelicensure nursing students' experiences of academic incivility during COVID-19: a qualitative inquiry. *Journal of Advanced Nursing*, *79*, 3370–3381. <https://doi.org/10.1111/jan.15656>
