# Supplementary material for: The Factors That Contribute to Dysfunctional Behavior in Active Military Personnel: An Umbrella Review
Source: Trauma Violence Abuse. 2025 Jul 12;27(4):1448–61. doi: 10.1177/15248380251349776 (PMC13578646; doi:10.1177/15248380251349776)
Supplement: Supplementary file 2 [file sj-docx-2-tva-10.1177_15248380251349776.docx]

# Supplemental Material 2

**Table S2**

Articles and Reasons for Full-Text Exclusion

| Author | Year | Article title | Journal title | Exclusion reason |
| --- | --- | --- | --- | --- |
| Al Alyani, O. et al. | 2023 | Review of the prevalence of opioid use disorder following traumatic brain injury | World Neurosurgery | No contributory factors discussed |
| Bayliss, L. T. et al. | 2022 | Suicide capability within the ideation-to-action framework: A systematic scoping review | PLOS One | Wrong outcomes - not dysfunctional behavior |
| Birkley, E. L. et al. | 2016 | Posttraumatic stress disorder symptoms, intimate partner violence, and relationship functioning: A meta-analytic review | Journal of Traumatic Stress | Does not distinguish active-duty military personnel from other populations |
| Bowen, G. L. et al. | 2017 | Preventing of family maltreatment in the U. S. Air Force: A systematic review of research on active-duty military personnel | Journal of Family Social Work | Wrong outcomes - not dysfunctional behavior |
| Brunet, A., & Monson, E. | 2014 | Suicide risk among active and retired Candian soldiers: The role of posttraumatic stress disorder | Canadian Journal of Psychiatry | Not a systematic review |
| Campbell, K. A. | 2023 | Dissociation: An overlooked concern in military training and an important factor in suciide | European Journal of Trauma and Dissociation | Not a systematic review |
| Campbell, W. R. et al. | 2017 | Primary care of men who have sex with men in the U.S. military in the post-don't ask, don't tell era: A review of recent progress, health needs, and challenges | Military Medicine | Not a systematic review |
| Caretta, R. F. et al. | 2023 | Gender differences in risks of suicide and suicidal behaviors in the USA: A narrative review | Current Psychiatry Reports | Not a systematic review |
| Castro, C. A. | 2014 | The US framework for understanding, preventing, and caring for the mental health needs of service members who served in combat in Afghanistan and Iraq: A brief review of the issues and the research | European Journal of Psychotraumatology | Not a systematic review |
| Castro, C. A. et al. | 2015 | Sexual assault in the military | Current Psychiatry Reports | Not a systematic review |
| Castro, C. A., & Kintzle, S. | 2014 | Suicides in the military: The post-modern combat veteran and the Hemingway effect | Current Psychiatry Reports | Not a systematic review |
| Cogan, A. M. et al. | 2020 | Gender differences in outcomes after traumatic brain injury among service members and veterans | American Academy of Physical Medicine & Rehabilitation | Not a systematic review |
| Ein, N. et al. | 2022 | The effects of child encounters during military deployments on the well-being of military personnel: A systematic review | European Journal of Psychotraumatology | Wrong outcomes - not dysfunctional behavior |
| Forbes, D. et al. | 2022 | Problematic anger in the military: Focusing on the forgotten emotion | Current Psychiatry Reports | Not a systematic review |
| Fralick, M. et al. | 2019 | Association of concussion with the risk of suicide: A systematic review and meta-analysis | JAMA Neurology | Does not distinguish active-duty military personnel from other populations |
| Galovski, T. E. et al. | 2022 | State of the knowledge of VA military sexual trauma research | Journal of General Internal Medicine | Not a systematic review |
| Godier-McBard, L. R., & Jones, M. L. | 2020 | Same-sex sexual violence in the military: A scoping review | Journal of Military, Veteran and Family Health | Not a systematic review |
| Goldbach, J. T., & Castro, C. A. | 2016 | Lesbian, Gay, Bisexual, and Transgender (LGBT) service members: Life after don't ask, don't tell | Current Psychiatry Reports | Not a systematic review |
| Gradus, J. L. | 2018 | Posttraumatic stress disorder and death from suicide | Current Psychiatry Reports | Not a systematic review |
| Greer, N. et al. | 2019 | Relationship of deployment-related mild traumatic brain injury to posttraumatic stress disorder, depressive disorders, substance use disorders, suicidal ideation, and anxiety disorders: A systematic review. | VA Evidence-based Synthesis Program Reports | Not a systematic review |
| Greer, N. et al. | 2018 | Outcomes associated with blast versus nonblast-related traumatic brain injury in US military service members and veterans: A systematic review | Journal of Head Trauma Review | Does not distinguish active-duty military personnel from other populations |
| Gromatsky, M. et al. | 2023 | The prevalence of nonsuicidal self-injury in military personnel: A systematic review and meta-analysis | Trauma, Violence, & Abuse | Does not distinguish active-duty military personnel from other populations |
| Heward, C. et al. | 2024 | A scoping review of military culture, military identity, and mental health outcomes in military personnel | Military Medicine | Not a systematic review |
| Indradevi, R. | 2016 | Toxic leadership over the years - A review | Purushartha | Not a systematic review |
| Irizar, P. et al. | 2021 | The prevalence of hazardous and harmful alcohol use across trauma-exposed occupations: A meta-analysis and meta-regression | Drug and Alcohol Dependence | Does not distinguish active-duty military personnel from other populations |
| Isaacs, J. Y. et al. | 2021 | Alcohol use and death by suicide: A meta-analysis of 33 studies | Suicide and Life-Threatening Behavior | Does not distinguish active-duty military personnel from other populations |
| Jacotte-Simancas, A. et al. | 2021 | Traumatic brain injury and the misuse of alcohol, opioids, and cannabis | International Review of Neurobiology | Not a systematic review |
| Khan, A. J. et al. | 2023 | A review of research on moral injury and suicide risk | Current Treatment Options in Psychiatry | Not a systematic review |
| Koenig, H. G. et al. | 2019 | Assessment of moral injury in veterans and active duty military personnel with PTSD: A review | Frontiers in Psychiatry | Not a systematic review |
| Kramer, E. B. et al. | 2020 | Use of the acquired capability for suicide scale (ACSS) among United States military and veteran samples: A systematic review | Journal of Affective Disorders | Wrong outcomes - not dysfunctional behavior |
| Kröger, C. et al. | 2023 | Hazing in the military: A scoping review | Journal of Military, Veteran and Family Health | Not a systematic review |
| Lacks, M. H. et al. | 2017 | A systematic review of the biopsychosocial-spiritual health of active duty women | Military Medicine | Wrong outcomes - not dysfunctional behavior |
| Lazar, S. G. | 2014 | The mental health needs of military service members and veterans | Psychodynamic Psychiatry | Not a systematic review |
| Lee, L. K. et al. | 2017 | Firearm laws and firearm homicides: A systematic review | JAMA Internal Medicine | No report of population |
| Lopes, J. et al. | 2023 | Adding insult to injury: Exploring the relation between moral injury and military sexual trauma | Journal of Military, Veteran and Family Health | Wrong outcomes - not dysfunctional behavior |
| MacGregor, J. C. D. et al. | 2021 | Intimate partner violence and work: A scoping review of published research | Trauma, Violence, & Abuse | Not a systematic review |
| Madsen, C. et al. | 2023 | Opioid prescribing and use in the Military Health System: A framework synthesis, FY2016-FY2021 | Pain Medicine | Not a systematic review |
| McDermott, M. C. et al. | 2024 | Female-specific health care of military female-designated service members and veterans: A systematic overview of reviews | Military Medicine | Not a systematic review |
| McIntire, K. L. et al. | 2021 | Factors increasing risk of suicide after traumatic brain injury: A state-of-the-science review of military and civilian studies | Brain injury | Not a systematic review |
| Miggantz, E. L. et al. | 2023 | Alcohol-involved sexual assault in the US military: A scoping review | European Journal of Psychotraumatology | Not a systematic review |
| Milner, A. J. et al. | 2016 | Does gender explain the relationship between occupation and suicide? Findings from a meta-analytic study | Community Mental Health Journal | Does not distinguish active-duty military personnel from other populations |
| Misca, G., & Forgey, M. A. | 2017 | The role of PTSD in bi-directional intimate partner violence in military and veteran populations: A research review | Frontiers in Psychology | Not a systematic review |
| Moradi, Y. et al. | 2021 | The global prevalence of depression, suicide ideation, and attempts in the military forces: A systematic review and meta-analysis of cross sectional studies | BMC Psychiatry | Does not distinguish active-duty military personnel from other populations |
| Morgan, L. | 2022 | Understanding sexual offences in UK military and veteran populations: Delineating the offences and setting research priorities | BMJ Military Health | Not a systematic review |
| Nazarov, A. et al. | 2015 | Role of morality in the experiences of guilt and shame within the armed forces | Acta Psychiatrica Scandinavica | Wrong outcomes - not dysfunctional behavior |
| Nelson, H. D. et al. | 2015 | Systematic review of suicide prevention in veterans | VA Evidence-based Synthesis Program Reports | Wrong outcomes - not dysfunctional behavior |
| Nir, I. | 2021 | Characteristics of suicidal male and female soldiers in various military forces | Journal of Mens Health | Not a systematic review |
| O'Leary, K. B., & Marcelli, M. | 2022 | Mental health outcomes among transgender veterans and active-duty service members in the United States: A systematic review | Federal Practitioner | Does not distinguish active-duty military personnel from other populations |
|  |  |  |  |  |
| Oliffe, J. L. et al. | 2021 | Segmenting or summing the parts? A scoping review of male suicide research in Canada | Canadian Journal of Psychiatry | Not a systematic review |
| Olliver, F. et al. | 2022 | Shame and guilt in the suicidality related to traumatic events: A systematic literature review | Frontiers in Psychiatry | Does not distinguish suicidal behavior from suicidal ideation |
| Osborne, A. K. et al. | 2022 | Military service and alcohol use: A systematic narrative review | Occupational Medicine | Not a systematic review |
| Peterson, A. et al. | 2022 | Those left behind: A scoping review of the effects of suicide exposure on veterans, service members, and military families | Death Studies | Not a systematic review |
| Ross, D. et al. | 2022 | Risk factors for mental ill health in UK army personnel: An overview | BMJ Military Health | Not a systematic review |
| Rozanov, V. | 2020 | Mental health problems and suicide in the younger generation - Implications for prevention in the Navy and merchant fleet | International Maritime Health | Not a systematic review |
| Sadler, N. et al. | 2021 | Suicide and suicidality in contemporary serving and ex-serving Australian Defence Force personnel | Australian & New Zealand Journal of Psychiatry | Not a systematic review |
| Salvatore, C., & Taniguchi, T. | 2021 | Military service and offending behaviors of emerging adults: A conceptual review | Social Sciences | Not a systematic review |
| Sharma, N. & Dhar, R. L. | 2022 | From curse to cure of workplace ostracism: A systematic review and future research agenda | Human Resource Management Review | Does not distinguish active-duty military personnel from other populations |
| Sher, L. | 2024 | Suicide in individuals with no psychiatric disorders: What makes you vulnerable? | QJM: An Internationa Journal of Medicine | Not a systematic review |
| Smith, N. B. et al. | 2014 | The role of trauma symptoms in nonsuicidal self-injury | Trauma, Violence, & Abuse | Not a systematic review |
| Stachow, E. | 2020 | Conflict-related sexual violence: A review | BMJ Military Health | Not a systematic review |
| Stander, V. A., & Thomsen, C. J. | 2016 | Sexual harassment and assault in the US military: A review of policy and research trends | Military Medicine | Not a systematic review |
| Stanley, E. A., & Larsen, K. L. | 2019 | Emotion dysregulation and military suicidality since 2001: A review of the literature | Political Psychology | Not a systematic review |
| Stevelink, S. A. et al. | 2014 | The prevalence of mental health disorders in (ex-)military personnel with a physical impairment: A systematic review | Occupational and Environmental Medicine | Not a systematic review |
| van den Berk Clark, C. et al. | 2018 | Women's health and the military | Primary Care - Clinics in Office Practice | No full-text version |
| Wadhawan, A. et al. | 2019 | Traumatic brain injury and suicidal behavior: A review | Journal of Alzheimers Disease | Not a systematic review |
| Weber, F. C. et al. | 2020 | Sleep disturbances and suicidality in posttraumatic stress disorder: An overview of the literature | Frontiers in Psychiatry | Not a systematic review |
| Whelpley, C. E., & McDaniel, M. A. | 2015 | Self-esteem and counterproductive work behaviors: A systematic review | Journal of Managerial Psychology | Does not distinguish active-duty military personnel from other populations |
| Williamson, V. et al. | 2018 | Occupational moral injury and mental health: Systematic review and meta-analysis | British Journal of Psychiatry | Wrong outcomes - not dysfunctional behavior |
| Willmund, G.-D. et al. | 2019 | German research perspectives on suicidality and the rationale for future multinational suicide prevention projects among military service personnel | International Review of Psychiatry | Not a systematic review |
| Wilson, L. C. | 2018 | The prevalence of military sexual trauma: A meta-analysis | Trauma, Violence, & Abuse | About experiences of military sexual trauma and not perpetration of MST |
| Xenakis, S. N. | 2016 | At risk for violence in the military | Psychiatric Clinics of North America | No full-text version |
| Mark, K. M. et al. | 2019 | The health and well-being of LGBTQ serving and ex-serving personnel: A narrative review | International Review of Psychiatry | Wrong outcomes - not dysfunctional behavior |
| Smith-Marek et al. | 2016 | Military/civilian risk markers for physical intimate partner violence: A meta-analysis | Violence and Victims | Does not distinguish active-duty military personnel from other populations |
| Bryan, C. J. et al. | 2015 | Combat exposure and risk for suicidal thoughts and behaviors among military personnel and veterans: A systematic review and meta-analysis | Suicide and Life-Threatening Behavior | Not a systematic review |
| Greer, N. et al. | 2019 | Prevalence and severity of psychiatric disorders and suicidal behavior in service members and veterans with and without traumatic brain injury: Systematic review | Journal of Head Trauma and Rehabilitation | Not a systematic review |
| Ramchand, R. et al. | 2015 | Prevalence of, risk factors for, and consequences of posttraumatic stress disorder and other mental health problems in military populations deployed to Iraq and Afghanistan | Current Psychiatry Reports | Not a systematic review |
| Livingston, W. S. et al. | 2023 | The association of military sextual harassment/assault with suicide ideation, plans, attempts, and mortality among US Service Members/Veterans: A meta-analysis | Trauma, Violence, & Abuse | Not a systematic review |
| Tam, C. C. et al. | 2020 | Prescription opioid misuse and its correlates among veterans and military in the United States: A systematic literature review | Drug and Alcohol Dependence | Not a systematic review |
| Brooks, S. K., & Greenberg, N. | 2018 | Non-deployment factors affecting psychological wellbeing in military personnel: Literature review | Journal of Mental Health | Not a systematic review |
| Cowlishaw, S. et al. | 2022 | Intimate partner violence (IPV) in military and veteran populations: A systematic review of population-based surveys and population screening studies | International Journal of Environmental Research and Public Health | No contributory factors discussed |
| MacManus, D. et al. | 2015 | Aggressive and violent behavior among military personnel deployed to Iraq and Afghanistan: Prevalence and link with deployment and combat exposure | Epidemiologic Reviews | Not a systematic review |
