# Supplementary material for: The Factors That Contribute to Dysfunctional Behavior in Active Military Personnel: An Umbrella Review
Source: Trauma Violence Abuse. 2025 Jul 12;27(4):1448–61. doi: 10.1177/15248380251349776 (PMC13578646; doi:10.1177/15248380251349776)
Supplement: Supplementary file 3 [file sj-docx-3-tva-10.1177_15248380251349776.docx]

# Supplemental Material 3

# Results of Quality Assessment

The quality of the included systematic reviews was assessed using the AMSTAR-2 checklist. There was a total of 16 items on the checklist (critical domains marked with an asterisk):

1. Did the research questions and inclusion criteria for the review include the components of PICO? (Yes/No)
2. *Did the report of the review contain an explicit statement that the review methods were established prior to the conduct of the review and did the report justify any significant deviations from the protocol? (Yes/Partial Yes/No)
3. Did the review authors explain their selection of the study designs for inclusion in the review? (Yes/No)
4. *Did the review authors use a comprehensive literature search strategy (Yes/Partial Yes/No)
5. Did the review authors perform study selection in duplicate? (Yes/No)
6. Did the review authors perform data extraction in duplicate? (Yes/No)
7. *Did the review authors provide a list of excluded studies and justify the exclusions? (Yes/Partial Yes/No)
8. Did the review authors describe the included studies in adequate detail? (Yes/Partial Yes/No)
9. *Did the review authors use a satisfactory technique for assessing the risk of bias (RoB) in individual studies that were included in the review? (Yes/Partial Yes/No)
10. Did the review authors report on the sources of funding for the studies included in the review? (Yes/No)
11. *If meta-analysis was performed, did the review authors use appropriate methods for statistical combination of results? (Yes/No/No meta-analysis conducted)
12. If meta-analysis was performed, did the review authors assess the potential impact of RoB in individual studies on the results of the meta-analysis or other evidence synthesis? (Yes/No/No meta-analysis)
13. *Did the review authors account for RoB in individual studies when interpreting/discussing the results of the review? (Yes/No)
14. Did the review authors provide a satisfactory explanation for, and discussion of, any heterogeneity observed in the results of the review? (Yes/No)
15. *If they performed quantitative synthesis, did the review authors carry out an adequate investigation of publication bias (small study bias) and discuss its likely impact on the results of the review? (Yes/No/No meta-analysis conducted)
16. Did the review authors report any potential sources of conflict of interest, including any funding they received for conducting the review? (Yes/No)

Table S2 presents the ratings of individual items and the overall assessment.

**Table S3**

Quality Assessment (AMSTAR-2) of The Included Systematic Reviews

| Citation | 1. | 2.* | 3. | 4.* | 5. | 6. | 7.* | 8. | 9.* | 10. | 11.* | 12. | 13.* | 14. | 15.* | 16. | Overall |
| --- | --- | --- | --- | --- | --- | --- | --- | --- | --- | --- | --- | --- | --- | --- | --- | --- | --- |
| Forkus et al. (2021) | Y | N | N | PY | Y | N | N | PY | N | N | N/A | N/A | N | Y | N/A | Y | Critically low |
| Fosse et al. (2019) | Y | N | N | PY | N | Y | N | PY | Y | N | Y | Y | Y | Y | Y | Y | Critically low |
| Hall et al. (2022) | N | N | N | PY | N | Y | N | PY | N | N | N/A | N/A | N | N | N/A | Y | Critically low |
| Holliday et al. (2020) | Y | PY | N | PY | Y | Y | N | Y | Y | N | N/A | N/A | Y | Y | N/A | Y | Low |
| Jamieson et al. (2023) | Y | Y | Y | PY | Y | Y | N | PY | Y | N | N/A | N/A | N | Y | N/A | Y | Critically low |
| Kelsall et al. (2015) | Y | N | N | PY | N | Y | N | PY | Y | N | Y | Y | Y | Y | Y | Y | Critically low |
| Kwan et al. (2020) | Y | PY | N | Y | N | N | N | PY | Y | N | Y | N | N | Y | N | Y | Critically low |
| McKenzie et al. (2023) | Y | N | N | PY | N | N | N | PY | Y | N | N/A | N/A | N | Y | N/A | Y | Critically low |
| Trevillion et al. (2015) | Y | PY | N | Y | N | N | N | PY | Y | N | N/A | N/A | Y | Y | N/A | Y | Low |
| Williamson et al. (2024) | Y | PY | N | Y | Y | N | N | PY | Y | N | N/A | N/A | N | Y | N/A | Y | Critically low |
| Yancey et al. (2024) | Y | N | N | PY | Y | N | N | N | N | N | N/A | N/A | N | Y | N/A | Y | Critically low |
